# Editorial: Interventional therapy of hepatocellular carcinoma

**DOI:** 10.3389/fmolb.2022.972207

**Published:** 2022-08-25

**Authors:** Jiansong Ji, Xiaoming Yang, Jianyuan Luo, Ivana Vucenik, Shuheng Jiang

**Affiliations:** ^1^ Lishui Central Hospital, Lishui, China; ^2^ Radiology, University of Washington, Seattle, WA, United States; ^3^ Health Science Centre, Peking University, Beijing, China,; ^4^ University of Maryland, School of Medicine, Baltimore, MD, United States; ^5^ Shanghai Cancer Institute, Shanghai, China

**Keywords:** HCC, Interventional therapy, treatment innovations, ACE, RFA—radiofrequency ablation

Globally, liver cancer is the most frequent fatal malignancy. In the United States, it ranks fifth (Sung et al., 2021). Patients are often diagnosed with liver cancer in advanced stages, contributing to its poor prognosis. Of all liver cancer cases, >90% are hepatocellular carcinomas (HCCs) (Sung et al., 2021). Currently, the incidence and mortality are increasing worldwide. Liver cancer is an extraordinarily heterogeneous malignant disease among the tumors that have so far been identified. HCC arises most frequently in the setting of chronic liver inflammation and fibrosis and takes a variety of course in individual patients to process to tumor. Because of the complex anatomy of the liver and association with underlying liver disease, management of these patients has been a challenge, considering both tumor and patient factors.

HCC classically is diagnosed at an advanced stage in a symptomatic patient, with very little therapeutic options. There is a rising incidence of HCC globally, the etiology of which includes viral hepatitis (B and C), alcohol, obesity, and dietary carcinogens, as the most common causes contributing to this high burden of HCC (Sung et al., 2021). Over the past 10 years, there has been considerable progress in the diagnosis and surgical treatment of HCC. Surgical resection is the best treatment option for early HCC. Unfortunately, majority of HCC patients already have advanced disease at the time of presentation. Therefore, liver cancer represents a major therapeutic challenge, and interventional therapy is the most used therapeutic approach today.

In this special issue, we present a summary on the most recent treatment modalities of HCC, using transcatheter arterial chemoembolization (TACE), radiofrequency ablation (RFA) and microwave ablation (MWA), while historical perspectives and the latest key findings are discussed. Comparing the efficacy and safety of TACE combining with apatinib (TACE-apatinib) and TACE-alone for patients with advanced HCC with hepatic arterioportal shunts (APS), it was concluded that TACE-apatinib was an efficacious and safe treatment for patients with advanced HCC with APS, and apatinib improved the efficacy of TACE in the treatment of these patients (Sun et al.). Retrospective study, investigating the efficacy and safety of sorafenib combined TACE (TACE + Sor) vs TACE combined with sorafenib plus immune checkpoint inhibitors (TACE + Sor + ICIs) in treating intermediate and advanced TACE-refractory HCC, showed that the therapeutic schedule of TACE + Sor + ICIs demonstrated efficacy and safety in intermediate and advanced TACE-refractory HCC (Zheng et al.). In a study to identify the independent risk factors for TACE refractoriness and to develop a novel TACE refractoriness score and nomogram for predicting TACE refractoriness in patients with HCC, the conclusion was that TACE refractoriness impaired the overall survival (OS) of HCC patients, that the number of tumors and bilobular invasion status were independent risk factors for TACE refractoriness, and that TACE refractoriness score could be an effective tool and easy approach to predict the risk of TACE refractoriness status (Chen et al.). A multicenter retrospective study was conducted with a purpose to use baseline variables to predict 1-year disease control for patients with HCC treated with TACE combined with sorafenib as initial treatment by applying a machine learning approach based on the random survival forest (RF) model (Zhong et al.). Because the RF model achieved a higher concordance index of 0.724 compared to that for the logistic regression model (0.709), it was concluded that the RF model was a simple and accurate approach for prediction of 1-year disease control for patients with HCC treated with TACE combined with sorafenib (Zhong et al.). The selection criteria for hepatic resection (HR) in intermediate-stage (IM) hepatocellular carcinoma (HCC) are still controversial. In a study conducted using the real-world data to evaluate the OS in treatment with HR or TACE, the conclusion was that HR was superior to TACE for intermediate-stage HCC in patients with LDH levels >192 U/L (Lu et al.). Investigating the predictive value of inflammatory biomarkers in patients with unresectable HCC for outcomes following the combination treatment of TACE plus sorafenib, the study indicated the prognostic value of quantitative inflammatory biomarkers in correlation with OS and progression-free survival (PFS) in unresectable HCC patients undergoing TACE plus sorafenib treatment (Zhang et al.). Trying to develop and validate a predictive model for early refractoriness of TACE in patients with HCC, an interesting multicenter retrospective study was conducted (Wang et al.), where a predictive model was established using forward stepwise logistic regression and nomogram (Wang et al.). Based on factors selected by logistic regression, a one-to-one propensity score matching (PSM) was conducted to compare PFS between patients who were present or absent of early TACE refractoriness. After PSM, the result showed that patients who were absent of early TACE refractoriness had a significantly higher PFS rate than those of patients who were present. This study presents a predictive model with moderate accuracy to identify patients with high risk of early TACE refractoriness, and patients with early TACE refractoriness may have a poor prognosis (Wang et al.). Trying to evaluate the safety and efficacy of TACE in elderly patients diagnosed as advanced HCC accompanied with different types of portal vein tumor thrombosis (PVTT), it was shown that palliative TACE treatment could be an accessible effective measure to improve the OS and PFS for both type I and type II PVTT patients (Tang et al.). Important study was conducted to establish a magnetic resonance imaging radiomics signature-based nomogram for predicting the progression-free survival of intermediate and advanced HCC patients treated with TACE plus RFA, showing that the radiomics signature was a prognostic risk factor, and a nomogram combined radiomics and clinical factors acted as a new strategy for predicted the PFS of intermediate and advanced HCC treated with TACE plus RFA (Fang et al.). Cases of HCC arising or involving the caudate lobe (HCC-CL) are relatively rare. It was shown that TACE treatment might be associated with better survival benefits in unresectable or “ablation unsuitable” HCC in the CL without macroscopic vascular invasion (MVI) and adequate liver function, compared with the non-selective TACE group, and should be considered as an important reliable therapy for surgeons and interventional radiologists (Yan et al.). To improve treatment of HCC, further research focused on the molecular mechanisms of HCC tumorigenesis is essential. The role of PTEN-Long, a translational variant of phosphatase and tensin homolog deleted on chromosome 10 (PTEN), a tumor suppressor frequently lost or mutated in several human tumors was evaluated in the development of liver cancer (Tan et al.). The study identified the antitumor function of PTEN-Long and suggested its potential role and utility for liver cancer treatment (Tan et al.).

In conclusion, with the understanding of the molecular mechanism of HCC and developing of new techniques, the theranostics of HCC have experienced innovations over the last few decades. The best treatment options which is interventional-based minimally invasive therapy have been discussed in this special issue. For example, Transarterial chemoembolization, Radiofrequency ablation, Microwave ablation. In recent years, new emerging interventional therapy has been developing towards non-invasive and intellectual development, playing an increasingly important role in the treatment of liver cancer. Additionally, multidisciplinary strategies for HCC treatment have been highly recommended by the clinical guidelines to further improve the survival and reduce the side-effect for HCC patients.
